# Study on prevalence of Fasciolosis in buffaloes at Anand and Ahmedabad districts, Gujarat, India

**DOI:** 10.14202/vetworld.2015.870-874

**Published:** 2015-07-14

**Authors:** Suchit S. Pandya, Jigar J. Hasnani, P. V. Patel, Vandip D. Chauhan, Nitin D. Hirani, Ravi Shukla, Hitesh B. Dhamsaniya

**Affiliations:** 1 Department of Veterinary Parasitology, College of Veterinary Science and Animal Husbandry, Anand Agricultural University, Anand, Gujarat, India; 2 Department of Livestock production and Management, College of Veterinary Science and Animal Husbandry, Anand Agricultural University, Anand, Gujarat, India; 3 Department of Animal Reproduction, Gynecology and Obstetrics, College of Veterinary Science and Animal Husbandry, Navsari Agricultural University, Navsari, Gujarat, India

**Keywords:** buffalo, Fasciolosis, fecal, liver, prevalence rate

## Abstract

**Aim:**

This study was undertaken to derive the prevalence rate of Fasciolosis in buffaloes by a collection of fecal and liver samples from Anand and Ahmedabad districts’ local slaughter houses.

**Materials and Methods:**

Fecal and liver samples were collected during ante- and post-mortem examination, respectively, and brought to the department laboratory preserved in 10% formalin for further processing. Fecal samples were processed with qualitative examination *viz*.; sedimentation technique for identification of the ova. Liver samples were also examined for the presence of gross parasites.

**Results:**

The highest prevalence rate was observed in the month of December (25.97% fecal and 33.33% liver samples) and lowest in the month of May (10.71% fecal and 11.76% liver samples) at Anand district. In the area of Ahmedabad district, the highest prevalence rate was recorded in the month of October and February (26.98%) and lowest in the month of May (10.34%) for the fecal and highest prevalence was observed in the month of February (26.98%) and lowest in May (11.11%) for the liver samples.

**Conclusion:**

It can be concluded that the heavy infection is present in Anand and Ahmedabad districts, especially in the month of winter followed by monsoon and the least in summer.

## Introduction

Buffaloes are the important multipurpose farm animals in the Indian sub-continent, contributing significantly to meat and milk production. Because of their habitats, they suffer from a wide variety of parasitic disease amongst which Fasciolosis is an important helminthic disease in India. Fasciolosis is one of the most serious and widely prevalent helminthic diseases caused by Fasciola hepatica in hilly tracks, and Fasciola gigantica in all other places. Fasciolosis in buffaloes is asymptomatic, subclinical, and/or chronic form of the disease, adversely affecting their reproductive cycle, weight gain, food conversion efficiency, and productivity. The frequency of outbreaks increases between October and May, but sporadic outbreaks continue throughout the year [1]. The host suffers from unnoticed ill effects of the disease for a prolonged period before the disease is detected at a veterinary clinic and/or at the abattoir [2].

Among different conditions, affecting mammary system, parasitic infections is one of the important conditions, which clinically appears as an excessive accumulation of fluid in the interstitial spaces [3]. In buffaloes, the adverse effect of Fasciolosis, in terms of reduction in milk yield, becomes more prominent during the latter stages of lactation. The milk loss in the state due to Fasciolosis has been observed highest for buffaloes, followed distantly by crossbred and indigenous cows. The annual milk loss in the state of Uttarakhand due to Fasciolosis has been estimated to be of 90.41 crores [4].

Both Anand and Ahmedabad districts possess well-irrigated area for the farming. Due to good irrigation facility, chances of water stagnation increases and it will increase the parasitic infection mainly Fasciolosis in animals. *Fasciola* spp. spread by *Lymnea* spp. snail which is well-grown in any water stagnated area.

Our main objectives of this study are to derive month wise, seasonal, and overall prevalence of this fluke. By using data of this study, prophylactic measures can be taken in future for protecting farm animals and loss of milk production, and economic losses can be saved of farm owners. In our study, both liver and fecal samples were taken because in sub-acute and chronic cases of Fasciolosis ova are not detected in fecal samples but immature stage of parasites are still present in body/liver of animals and damage it and cause production loss.

## Materials and Methods

### Ethical approval

Present study was conducted after necessary approval from advisory and institutional animal ethics committee of Anand Veterinary College.

### Study area and sample collection

The study was carried out to ascertain the prevalence aspect of Fasciolosis in slaughtered buffaloes at Anand (22.5560°N, 72.9510°E) and Ahmedabad (72.5800°E, 23.0300°N) districts’ local slaughter houses of Gujarat. Both Anand and Ahmedabad districts have well canal irrigated area, so chances of occurrence of parasitic disease in farm animals are more and due to this, milk production decrease in animals. Hence, for prophylactic measures epidemiological study is needed. Sometimes during fecal sample examination, false negative result happens, and the animal undergoes false diagnosed, so in this study we have collected both fecal and liver samples because larval stage was also detected in the liver of animals. The study was undertaken for the period of 12 months from March-2013 to February-2014. For findings of prevalence, the slaughtered buffaloes were examined for the presence of *Fasciola* spp. in their liver and bile duct during post-mortem examination and fecal samples and intestinal contents were also collected for the detection and identification of ova of *Fasciola* spp. during ante-mortem and post-mortem examination, respectively. During whole of the study, the samples were collected during morning hours from the Anand district (local Anand city slaughter house and Kanjari slaughter house) and in evening hours from Ahmedabad district (Jamalpur and Mirzapur Ahmedabad Municipal Corporation slaughter houses) and samples were collected in small and clean sterilized polythene bags and preserves in 10% formalin. The bags were numbered, ligated with rubber bands, and were brought to the laboratory for further processing and examination for the presence of parasitic infection.

### Processing of fecal samples

Fecal samples were processed by qualitative examination *viz.;* sedimentation technique for the identification of the ova in the laboratory [5].

### Processing of liver and bile ducts

Liver and bile ducts were examined at the slaughter houses only for the detection of the presence of mature and immature stages of *Fasciola* spp. Infected liver and bile ducts were brought to the department laboratory preserved in 10% formalin. Parasites were collected from liver and bile duct for further identification of the species of the parasites.

### Meteorological data

In this study, November, December, January, and February were considered as winter, March, April, May, June as summer, and July, August, September, and October were considered as monsoon seasons. Data were collected from Department of Agricultural Meteorology, BACA, AAU, Anand for the study period of March-2013 to February-2014. It is shown that the relative humidity is quite higher in the winter season also comparing to previous years that is average 90% in winter, 77% in summer, and 93% in the monsoon season. Humidity has a direct relation to the growth of the parasites.

## Results

**Figure-1:**
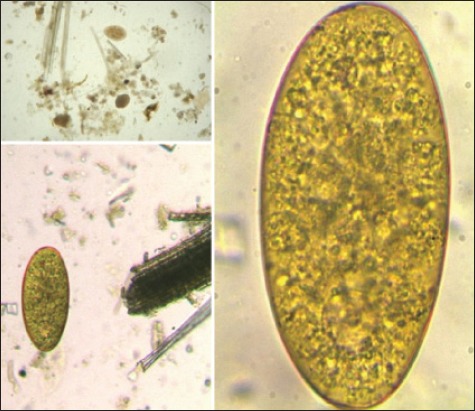
Ova of *Fasciola gigantica* in a smear of fecal sample (×10).

**Figure-2:**
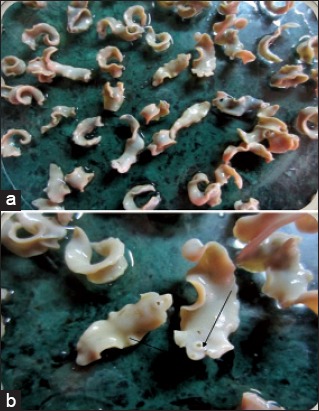
(a) Gross specimen of *Fasciola gigantica* (b) oral and ventral sucker of *Fasciola gigantica*.

Ova were identified as of *Fasciola* spp. by sedimentation technique under the microscope in the laboratory on bases of its morphological characteristics (Figure-1). Species of the collected parasites were identified as *Fasciola gigantica*, on the bases of its morphological characteristics, that is broad shoulder and size of the parasite (Figure-2). A total of 760 and 792 fecal samples and 701 and 743 liver samples were collected, of which 151 (19.87%) and 163 (20.58%) fecal samples and 164 (23.39%) and 157 (21.13%) liver samples were found positive for the *Fasciola* spp. infection from Anand and Ahmedabad districts, respectively (Table-1).

**Table-1:** Overall prevalence of *Fasciolosis* in slaughtered buffaloes at Anand and Ahmedabad Districts.

Samples	Anand district (%)	Ahmedabad district (%)
Fecal samples		
Total examined	760	792
Total positive	151 Summer-29 Monsoon-53 Winter-69	163 Summer-42 Monsoon-57 Winter-69
Summer	12.83	16.93
Monsoon	21.54	21.83
Winter	23.95	22.61
Prevalence	19.87	20.58
Liver		
Total examined	701	743
Total positive	164 Summer-31 Monsoon-56 Winter-77	157 Summer-41 Monsoon-55 Winter-61
Summer	12.34	17.60
Monsoon	24.34	22.08
Winter	28.62	23.37
Prevalence	23.39	21.13
Overall prevalence district wise		
Total sample, Examined	1461, (liver+fecal)	1535, (liver+fecal)
Total positive	315	320
Prevalence	21.70	20.84
Overall prevalence of both the districts		
Total sample (Anand+Ahmedabad)	Total positive (Anand+ Ahmedabad)	Overall prevalence
2996	635	21.19

### Seasonal prevalence

A total of 151 and 163 samples were found positive out of 760 and 792 samples of Anand and Ahmedabad districts, respectively. In Anand district, seasonal prevalence found to be 12.83% (29), 21.54% (53), and 23.95% (69), and in Ahmedabad district it is found to be 16.93% (42), 21.83% (57), and 22.61% (69) of summer, monsoon, and winter, respectively (Table-1), by fecal sample collection. Seasonal prevalence also derived by liver sample collection. In Anand district, seasonal prevalence found to be 12.34%, 24.34%, and 28.62%, and in Ahmedabad district it is found to be 17.60%, 22.08%, and 23.37% of summer, monsoon, and winter, respectively (Table-1).

### Month wise and overall prevalence

The highest prevalence rate was observed in the month of December 25.97% and lowest in the month of May 10.71% for the fecal samples and for the liver samples, prevalence rate was recorded highest in the month of December 33.33% and lowest in May 11.76% at Anand district. At Ahmedabad district the prevalence was observed highest in month of October 26.98% and same prevalence in the month of February 26.98% and lowest in the month of May 10.34% for the fecal samples and for the liver samples, prevalence rate was recorded highest in month of February 26.98% and lowest in May 11.11%. Hence. from August onward the intensity of the infection is increased up to the month of February and from March onward intensity of the infection is decreased up to the month of July.

### Overall prevalence

Overall prevalence in Anand and Ahmedabad districts was found to be 21.70% and 20.84%, respectively. Overall prevalence from both districts combinely found to be 21.19% (Table-1).

## Discussion

In the present study, seasonal prevalence found to be highest in winter because of availability of humid climate, due to extended rainfall in winter season followed by monsoon, and the least in summer due to unavailability of favorable climate for survival of parasite. Similar findings were documented by Maqbool *et al*. [6] who noted the highest prevalence in monsoon 24.00% in comparison to winter 20.00% and lowest in summer 9.0%, Garg *et al*. [7] reported highest prevalence in winter 15.57% followed by summer and monsoon, Khan *et al*. [8] encountered the highest prevalence in winter 39.08%, followed by monsoon 20.33%, and summer 12.92%. Affroze *et al*. [9] reported significantly higher prevalence of Fascioliasis in winter season (51.33%), followed by rainy (24.24%), and summer season (18.10%) which are in line with the present study. Lifecycle of *Fasciola* spp. starts in monsoon season and continues till winter season. However, as compared to the present findings, a comparatively higher and contradictory prevalence were recorded by some authors *viz*., Kuchai *et al*. [10] reported highest seasonal prevalence 45.19% during wet season while as only 24.40% was recorded during the dry season in cattle. Various other studies reported less prevalence rate compared to the present study. Yadav *et al*. [11] revealed 4.03% infection in summer, followed by 3.24% in winter, and 1.01% infection in the rainy season.

Month wise prevalence were recorded by Yadav *et al*. [12] documented higher prevalence 15.4% in the month of January, Qureshi *et al*. [1] noted highest prevalence in September 32.33%, and the lowest in May 4.83%. In the present study, higher prevalence occur in the months of January and February because the monsoon season was extended up to the month of January, so availability of metacercarae infected grasses becomes more and due to this, more animals are getting infected with the Fasciolosis. It may be one of the reasons of global warming that during the study period, there was abnormal weather and rain during this period. Rehman *et al*. [13] documented maximum prevalence from January to September, while minimum prevalence was observed from October to December, which shows quite opposite result to the present study. Various studies in relation to the overall prevalence of Fasciolosis have been performed and have provided evidence in support of the result *viz*., Maqbool *et al*. [6] noted 25.59%, 26.16%, 13.7%, and 10.5% prevalence in slaughtered buffaloes, buffaloes at livestock farms, veterinary hospitals, and in household buffaloes, respectively, with the overall prevalence of 24.00%, Mungube *et al*. [14] reported 26.0% liver condemnation rate by *F. gigantica*, Gupta *et al*. [15] recorded 23.8% buffaloes infected with *F. gigantica* by doing fecal examination of buffaloes. Similar findings were also recorded by Khan *et al*. [8] who documented 22.4% prevalence in Punjab, Kuchai *et al*. [10] reported 27.69% and 21.91% prevalence in cattle of livestock farm and household, respectively, Qureshi *et al*. [1] reported 14.69% prevalence, which is completely in line with the present study. However, as compared to the present findings, a comparatively higher prevalence were recorded by Gupta *et al*. [15] found 37.5% buffaloes infected by doing necropsy examination and 35.0% buffaloes infected by doing fecal sample examination at Lucknow while 32.4% buffaloes found infected at Bareilly, Garg *et al*. [7] noted 31.14% prevalence in buffaloes at abattoirs of North-India. This variation can probably be attributed to the less intensification of veterinary and extension services in the study area and also due to the favorable weather condition for the parasite. In the current study, the prevalence of Fasciolosis in buffaloes by post-mortem examination is 23.39% and 21.13% at Anand and Ahmedabad districts, respectively, while Abdulhakim and Addis [16] reported 39.8% prevalence in the slaughtered buffaloes at Ethiopia, Affroze *et al*. [7] recorded 31.14% prevalence in buffaloes and Maqbool *et al*. [17] noted 25.40% prevalence in slaughtered cattle. This difference occurs may be due to more availability of cultivated grasses or water stagnated area at the study sites. Some other studies also show lower prevalence rate of Fasciolosis in buffaloes as compared to the present study. Yadav *et al*. [11] recorded 4.27% prevalence of Fasciolosis by fecal sample examination while 12.09% prevalence found in slaughtered buffaloes at national capital region Delhi, Gupta *et al*. [15] reported 7.7% cattle, 4.6% buffaloes, and 1.7% buffaloes showed *F. gigantica* infection by fecal sample examination at Gorakhpur, Agra, and Varanasi, respectively, while during necropsy examination they found 9.1% and 8.1% prevalence at Varanasi and Allahabad, respectively, Garg *et al*. [7] reported 13.9% buffaloes infected with *Fasciola* spp. In the present result, the variation in the finding with the above-mentioned authors might be due to the differences in the sample size, species variation, browsing habit, and also due to the difference in the climatic condition of individual area. It may be due to the less rainfall in the studied area and because of that, less availability of grasses and the metacercarae, which was encysted on those grasses. In the present study, higher prevalence were noted in winter, it may be due to higher humidity recorded in the winter season, which favors the growth of the parasites.

## Conclusion

Though good managemental practices established, this study revealed the higher intensity of the infection in buffaloes. Infection is increased from August onward and it is increased up to the month of February and from March onward intensity of the infection is decreased up to the month of July. Winter season was found critical for the animals because the highest infection noticed followed by monsoon and summer seasons. These occurred because the highest humidity was recorded in winter season comparing to previous years which is the ideal favorable survival condition for this parasite due to the extended rainfall in the winter season. The overall prevalence found to be higher in Anand compared to the Ahmedabad district because of more availability of canal irrigated areas.

## Authors’ Contributions

This study is the major component of the work toward the M.V.Sc thesis of the first author SSP, under the guidance of JJH and PVP. VDC and RS helped in sample collection from various abattoirs. HBD helped in processing of all the collected samples. NDH helped in identification of ova and species of the parasites and thoroughly revised the manuscript. All authors have read and approved the final version of the manuscript.
